# Trombo Atrial Esquerdo e Contraste Espontâneo Denso no Uso de Anticoagulante Oral de Ação Direta em Fibrilação Atrial: Visão de Centro Referenciado

**DOI:** 10.36660/abc.20210658

**Published:** 2022-09-15

**Authors:** Thiago Marques, Francisco Darrieux, Fábio Gouvêa, Leandro Garambone, Ana Paula Lindoso, João Lage, Luciana Sacilotto, Ana Lúcia Coimbra, Martina Pinheiro, Natália Olivetti, Sissy Lara, Carina Hardy, Guilherme Athayde, Denise Hachul, Cristiano Pisani, Tan Chen Wu, Maurício Scanavacca

**Affiliations:** 1 Hospital das Clínicas Faculdade de Medicina Universidade de São Paulo São Paulo SP Brasil Instituto do Coração, Hospital das Clínicas , Faculdade de Medicina , Universidade de São Paulo , São Paulo , SP – Brasil

**Keywords:** Fibrilação Atrial, Cardioversão Elétrica, Ecocardiografia Transesofágica

## Abstract

**Fundamento:**

No tratamento da fibrilação atrial (FA), a arritmia sustentada mais frequente, com ablação por cateter (ABL) ou cardioversão elétrica (CVE), o período periprocedimento é uma das fases mais críticas. Atualmente, o uso de novos anticoagulantes orais de ação direta (DOAC) é cada vez mais frequente, no entanto, no mundo real, ainda existem poucos dados de estudos sobre a incidência de trombo no átrio esquerdo (TrAE) ou contraste espontâneo denso (CE) no ecocardiograma transesofágico (ETE).

**Objetivo:**

Analisar a prevalência de TrAE, por ETE, em pacientes em uso de DOAC submetidos à CVE/ABL. Secundariamente: avaliar a associação de comorbidades com a presença de trombos e CE.

**Métodos:**

Estudo de coorte retrospectivo, unicêntrico, com pacientes do Ambulatório de Arritmia (InCor-HCFMUSP). Foram selecionados e analisados dados clínicos e ecocardiográficos no prontuário da instituição de pacientes com indicação de procedimentos e em uso de DOACs. Considerado um nível de significância de 5%.

**Resultados:**

Foram incluídos 354 pacientes, no total de 400 procedimentos, de março de 2012-março de 2018. TrAE foi encontrado em 11 pacientes (2,8%), associado com idade avançada (p=0,007) e CHA2DS2-VASc maior (p<0,001). Foi encontrado CE no AE no procedimento antes da ETE em 29 pacientes (7,3%), com menor FEVE (p <0,038) e maior dimensão do AE (p <0,0001).

**Conclusão:**

A incidência de TrAE e CE em pacientes em uso de DOAC no contexto de CVE/ABL de FA, embora pequena, não é desprezível. Pacientes com escore CHA2DS2-VASc maior, principalmente mais idosos e com diâmetro do AE maior, são mais propensos a esses achados ecocardiográficos.

## Introdução

A fibrilação atrial (FA) é a arritmia sustentada mais frequente na prática clínica, com prevalência em torno de 1% na população geral. ^1^ Uma das fases mais críticas no tratamento da FA refere-se ao período periprocedimento da ablação por cateter (ABL) ou cardioversão elétrica (CVE), onde o risco de um evento tromboembólico deve ser minimizado com o uso de anticoagulação oral. No passado, apenas a varfarina estava disponível, com uma taxa de incidência periprocedimento de eventos tromboembólicos variando entre 0,5 e 1,6%. ^2 , 3^ A prevalência de trombo no átrio esquerdo (TrAE) varia entre 0,6% e 6,4% em pacientes em tratamento com varfarina. ^4 - 6^ Atualmente, o uso de novos anticoagulantes orais de ação direta (DOACs) é cada vez mais frequente; no entanto, em condições do mundo real, ainda existem poucos dados de estudos sobre a incidência de trombo ou contraste espontâneo denso na ecocardiograma transesofágico (ETE) no AE. ^7 - 9^

## Métodos

### População estudada

Estudo de coorte retrospectiva, unicêntrico com pacientes acompanhados no Ambulatório de Arritmia do Instituto do Coração – Hospital das Clínicas da Faculdade de Medicina da Universidade de São Paulo (InCor-HCFMUSP).

Pacientes com idade ≥ 18 anos com diagnóstico de FA persistente, submetidos a CVE e/ou ABL, em uso de DOACs pelo menos 3 semanas antes do procedimento, foram incluídos, consecutivamente, por um período de 6 anos (2012-2018) Todos os pacientes foram submetidos a ETE na mesma internação. A escolha e a dose de DOAC (rivaroxabana, dabigatrana ou apixabana) foram estabelecidas pelo médico assistente com avaliação do clearance de creatinina durante o acompanhamento clínico. Pacientes que realizaram o procedimento (cardioversão ou ablação) há mais de 6 meses puderam ser incluídos novamente. Os pacientes mantiveram o uso constante do DOAC, suspendendo apenas no dia do procedimento.

Foram analisados dados clínicos como idade, comorbidades e escore CHA2DS2-VASc, bem como dados estruturais do ETE – dimensão do átrio esquerdo (AE) e fração de ejeção do ventrículo esquerdo (FEVE). A dimensão do diâmetro foi escolhida ao invés do volume indexado do AE, pois esta medida não é padrão no exame. A equipe da ETE definiu a presença de trombos e/ou contraste espontâneo denso.

## Objetivos

O objetivo primário foi analisar a prevalência de trombo em AE, por meio do ETE, em pacientes em uso de DOAC por pelo menos 3 semanas, submetidos à CVE e/ou ablação. O objetivo secundário foi avaliar a associação de comorbidades com a presença de trombos e contraste espontâneo.

Este estudo faz parte de um registro maior (“Registro Institucional com o uso dos Anticoagulantes de ação direta em pacientes com Fibrilação atrial não-valvar”), CAAE Nº 57417716.6.0000.0068, aprovado pelo Comitê Institucional de Ética em Pesquisa do Hospital das Clínicas, Universidade de São Paulo – CEP-HCFMUSP, aprovação nº 1.637.837. O Comitê de Ética concordou com a não necessidade de um consentimento informado, já que o estudo foi retrospectivo, em dados de prontuários da instituição. Todos os dados foram anonimizados antes de serem coletados.

### Análise estatística

Os dados coletados foram descritos por meio de média e desvio padrão para variáveis contínuas de distribuição normal. As variáveis categóricas foram descritas em números absolutos e porcentagens. A normalidade das variáveis foi testada pelo teste de Kolmogorov-Smirnov. Os testes estatísticos foram realizados, de acordo com o tipo de variável (qualitativa/quantitativa) e a normalidade de distribuição, usando teste t de Student não pareado, teste do qui-quadrado e regressão logística multivariada. Valores de p menores que 0,05 foram considerados estatisticamente significativos. A análise estatística foi realizada por meio do software SSPS (versão 22.0).

## Resultados

Foram incluídos 354 pacientes, de março de 2012 a março de 2018. Foram realizados 400 procedimentos, 122 cardioversões e 278 ablações. Dentre as comorbidades observadas, a maioria dos pacientes apresentava hipertensão essencial e cerca de 33% eram acima de 60 anos ( Tabela 1 ). Houve diferença entre os grupos que fizeram uso de DOACs, com maior presença de AVC prévio nos pacientes que usaram dabigatrana. ( Tabela 2 )

**Tabela 1:** Características da população

Sexo masculino (n) (%)		288 (72)
Idade (DP)		59,9 (11,4)
FEVE % (DP)		59,6 (8,5)
AE mm (DP)		43,3 (6,2)
CHA _2_ DS _2_ -VASc (DP)		1,68 (0,25)
Hipertensão (n) (%)		214 (53,6)
AVE (n) (%)		27 (6,8)
IC (n) (%)		48 (12,0)
Diabetes (n) (%)		61 (15,3)
Vasculopatia (n) (%)		26 (6,5)
DOACs	Apixabana (n) (%)	79 (19,8)
	Dabigatrana (n) (%)	99 (24,8)
	Rivaroxabana (n) (%)	222 (55,5)
Dosagem – dose única (n) (%)		222 (55,5)
Trombo (n) (%)		11 (2,8)
Contraste espontâneo (n) (%)		29 (7,3)
Trombo e contraste (n) (%)		39 (9,8)
Procedimento	Ablação (n) (%)	278 (69,5)
	Cardioversão (n) (%)	122 (30,5)

*DOAC: Direct-acting oral anticoagulants [anticoagulantes orais de ação direta; AE: átrio esquerdo; FEVE: fração de ejeção do ventrículo esquerdo; AVE: acidente vascular encefálico; IC: insuficiência cardíaca.*

**Tabela 2:** Características dos pacientes de acordo com DOACs

	DOACs			p

	Apixabana n: 79	Dabigatrana n: 99	Rivaroxabana n: 222	
Sexo masculino (%)	61 (77,2)	66 (66,7)	161 (72,5)	0,29
IC (%)	5 (6,3)	15 (15,2)	28 (12,6)	0,18
AVE (%)	5 (6,3)	13 (13,1)	9 (4,1)	**0,011**
Hipertensão (%)	35 (44,3)	58 (58,6)	121 (54,5)	0,15
Diabetes (%)	8 (10,1)	18 (18,2)	35 (15,8)	0,32
Vasculopatia (%)	5 (6,3)	4 (4)	17 (7,7)	0,48
Trombo (%)	4 (5,1)	3 (3,0)	4 (1,8)	0,31
Contraste espontâneo (%)	2 (2,5)	13 (13,1)	14 (6,3)	**0,018**

*Teste qui-quadrado. AVE: acidente vascular encefálico; IC: insuficiência cardíaca; DOAC: Direct-acting oral anticoagulant (anticoagulantes orais de ação direta).*

Houve presença de trombo em AE em 11 pacientes ( Tabela 3 ), de um total de 400 procedimentos (2,8%), e associação com idade avançada (p = 0,007) e maior CHA2DS2-VASc (p <0,001) foi observado. ( Tabela 4 )

**Tabela 3:** Tipo e dosagem de DOAC em pacientes com trombo de átrio esquerdo

Paciente N°	Sexo	Idade	CHA2DS2-VASc	Ritmo no ETE	DOAC
1	Feminino	72	2	Irregular	Rivaroxabana 20mg
2	Feminino	67	3	Irregular	Dabigatrana 150mg
3	Masculino	74	3	Irregular	Rivaroxabana 20mg
4	Masculino	66	1	Irregular	Apixabana 2,5mg
5	Masculino	64	1	Irregular	Apixabana 5mg
6	Masculino	67	5	Irregular	Rivaroxabana 20mg
7	Feminino	83	4	Irregular	Dabigatrana 110mg
8	Masculino	58	3	Irregular	Apixabana 5mg
9	Feminino	78	6	Irregular	Dabigatrana 110mg
10	Feminino	75	3	Irregular	Rivaroxabana 20mg
11	Masculino	56	2	Irregular	Apixabana 5mg

*ETE: ecocardiograma transesofágico;*

**Tabela 4:** Análise dos fatores de risco em relação à presença de trombo e contraste espontâneo

	Trombo		p	Contraste espontâneo		p	Trombo e contraste		p
		
	Sim	Não		Sim	Não		Sim	Não	
Sexo masculino (%)	6 (2,1)	282 (97,9)	0,19	18 (6,3)	270 (93,8)	0,22	24 (8,3)	264 (91,7)	0,13
Idade (DP)	69,1 (8,2)	59,6 (11,4)	**0,007**	61,7 (7,2)	59,7 (11,7)	0,19	63,5 (8,1)	59,5 (11,7)	**0,036**
IC (%)	2 (4,2)	46 (95,8)	0,52	6 (12,5)	42 (87,5)	0,14	8 (16,7)	40 (83,3)	0,09
AVE (%)	3 (11,1)	24 (88,9)	**0,006**	6 (22,2)	21 (77,8)	**0,002**	9 (33,3)	18 (66,7)	**<0,001**
Hipertensão (%)	8 (3,7)	206 (96,3)	0,20	17 (7,9)	197 (92,1)	0,57	25 (11,7)	189 (88,3)	0,16
Diabetes (%)	1 (1,6)	60 (90,4)	0,56	7 (11,5)	54 (88,5)	0,17	8 (13,1)	53 (86,9)	0,34
Vasculopatia (%)	1 (3,8)	25 (96,2)	0,72	0 (0)	26 (100)	0,14	1 (3,8)	25 (96,2)	0,29
Dose duas vezes ao dia (%)	7 (3,9)	171 (96,1)	0,20	15 (8,4)	163 (91,6)	0,42	22 (12,4)	156 (87,6)	0,12
AE (DP)	43,6 (4,4)	43,3 (6,2)	0,86	47,8 (5,6)	43,0 (6,1)	**<0,001**	46,6 (5,6)	43,0 (6,1)	**<0,001**
FEVE (DP)	59,8 (10,0)	59,6 (8,5)	0,94	56,5 (9,0)	59,9 (8,4)	**0,038**	57,2 (9,3)	59,9 (8,4)	0,06
CHA _2_ DS _2_ -VASc (DP)	3,0 (1,5)	1,64 (1,3)	**0,001**	2,1 (1,4)	1,6 (1,4)	0,06	2,4 (1,5)	1,6 (1,3)	**0,001**

*Teste qui-quadrado. AE: átrio esquerdo; AVE: acidente vascular encefálico; FEVE: fração de ejeção do ventrículo esquerdo; IC: insuficiência cardíaca*

Contraste espontâneo denso em AE no procedimento prévio à ETE foi observado em 29 pacientes (7,3%). Comparando os dados desse grupo com aqueles sem a presença de contraste, observou-se menor FEVE (p <0,038) e maior dimensão do AE (p <0,0001). ( Tabela 4 )

A presença de achados combinados (trombo e contraste) ocorreu em 39 (9,8%) pacientes. Nesse grupo, observou-se maior idade (p = 0,007), maior dimensão do AE (p <0,001) e maior CHA2DS2-VASc (p <0,001). ( Tabela 4 )

Ao combinar a influência de cada fator por meio de regressão logística múltipla, há um risco maior em pacientes com AVE prévio (OR: 4,8), em pacientes com IC (OR: 2,9), em idosos (OR: 1,04) e naqueles com tamanho atrial maior (OR: 1,11). Uma análise adicional foi realizada entre os pacientes que usaram DOAC em dose única diária e aqueles que usaram a medicação duas vezes ao dia, sem diferença entre os grupos quanto a trombo, contraste ou ambas as situações combinadas. ( Tabela 5 )

**Tabela 5:** Análise multivariada dos fatores de risco

	OR	(95% IC)		p

		Inferior	Superior	
Sexo masculino	0,61	0,27	1,36	0,23
IC	2,90	1,15	7,31	**0,024**
Hipertensão	1,43	0,67	3,07	0,36
Idade (anos)	1,04	1,00	1,08	**0,045**
AVE	4,80	1,84	12,51	**0,001**
Dose (2x)	1,72	0,83	3,56	0,14
AE (mm)	1,11	1,04	1,17	**0,001**

*Regressão logística múltipla. AVE: acidente vascular encefálico; IC: insuficiência cardíaca; AE: átrio esquerdo; IC: intervalo de confiança.*

## Discussão

Na literatura atual, a prevalência de TrAE entre pacientes que foram adequadamente anticoagulados com varfarina antes da ETE varia de 0,3% a 7,7%, em comparação com 2,75% em outras séries com o uso de DOACs. ^10 - 14^

Nosso estudo demonstrou que, mesmo em pacientes em uso de DOACs, foi encontrada uma taxa (9,8%) de trombo ou contraste espontâneo denso no AE em pacientes submetidos à CVE eletiva periprocedimento e/ou ABL. Foi utilizado um período mínimo de 3 semanas de uso prévio de DOACs, que consideramos razoável baseado na literatura, e não houve diferença estatística comparado com 4 semanas.

Em uma análise de subgrupo do estudo RE-LY, as taxas de trombos no AE em pacientes antes da cardioversão foi de 1,5% para pacientes em uso de dabigatrana. No estudo ARISTOTLE, os registros de ETE estavam disponíveis em 86 pacientes em uso de apixabana e nenhum desses pacientes apresentava TrAE. No estudo ROCKET-AF, os dados de ETE não foram coletados para avaliar a prevalência de TrAE. ^15 - 17^

Frenkel et al. analisaram retrospectivamente dados de 388 pacientes com ETE antes da CVE de FA ou com flutter atrial, em uso de DOAC contínuo ou terapia com varfarina por 4 semanas. A prevalência de TrAE foi de 4,4% no grupo DOAC e 2,9% no grupo da varfarina, sem significância estatística. ^18^ Al Rawahi et al., analisando dados de 401 pacientes submetidos à ablação ou CVE, encontraram TrAE em 11,2% da amostra. Quando separamos os pacientes que usaram apenas DOACs, a presença de trombo nos que usaram dabigatrana, rivaroxabana e apixabana foi de 5%, 4% e 9%, respectivamente. ^19^ Wu et al., analisando 609 pacientes em uso de DOAC, com tempo médio de anticoagulação de 12 semanas, encontraram 17 pacientes (2,8%) com TrAE e 15 pacientes (2,5%) com contraste espontâneo no ETE, números comparáveis aos nossos achados. ^20^

Apesar das vantagens teóricas do tratamento de pacientes com FA com DOACs em relação à varfarina, no que diz respeito a questões de dosagem e anticoagulação subterapêutica, a prevalência de TrAE não é desprezível, mesmo com o paciente usando DOAC corretamente por pelo menos 3 semanas. Nossas taxas são comparáveis às relatadas anteriormente entre pacientes em uso de terapia anticoagulante com varfarina, que variam amplamente, conforme mencionado anteriormente.

Atualmente, a indicação de realização de ETE eletivo antes da ablação e/ou cardioversão da FA/flutter atrial ainda é controversa. De acordo com o Consenso HRS/EHRA/ECAS de 2017 sobre ABL de FA, 50% dos membros do grupo de escrita realizaram ETE de rotina, enquanto o grupo restante realizou ETE apenas se os pacientes tivessem fatores de risco significativos para trombo de AE ou não tivessem feito anticoagulação terapêutica por pelo menos quatro semanas. No entanto, é geralmente aceito que a presença de um trombo detectado por ETE é uma contraindicação para cardioversão ou ABL de FA.

Existem várias razões potenciais para a presença desses achados, apesar da anticoagulação eficaz com DOAC, que inclui fatores subjacentes do paciente, incluindo: miopatia atrial grave, que torna o trombo refratário; má adesão; dosagem inadequada devido a flutuações na depuração da droga (por exemplo, alterações na função renal) e níveis séricos inadequados da droga devido ao modo incorreto de administração (por exemplo, não tomar rivaroxabana com alimentos, levando a biodisponibilidade reduzida). É importante notar que não houve diferença entre os pacientes de acordo com o regime de dosagem do DOAC. Um outro fator que pode influenciar são as interações medicamentosas, no entanto, no nosso trabalho, não houve relato do uso concomitante de drogas com elevada interação já descritas pela literatura como, por exemplo, rifampicina, antirretrovirais e/ou antifúngicos, dentre outras.

Nosso estudo sugere que a triagem com ETE deve ser realizada em pacientes com altos escores CHA2DS2-VASc, AVC isquêmico prévio e dimensão do AE maior que 45 mm, apesar da terapia ininterrupta com DOAC, uma hipótese que deve ser testada em futuros ensaios clínicos randomizados.

### Limitações

Nosso estudo tem várias limitações. Primeiro, este é um estudo observacional transversal. Embora pacientes com história documentada de perda de doses de DOAC nas 3 semanas anteriores à ETE em nosso estudo tenham sido excluídos, não foi possível garantir a adesão total em todos os indivíduos. Além disso, não verificamos sistematicamente se os pacientes estavam tomando DOAC corretamente (por exemplo, administração de rivaroxabana com as refeições). No entanto, nosso desenho de estudo reflete a prática do mundo real. Outra limitação refere-se ao fato de não ter sido possível descartar se parte desses pacientes já apresentava trombos no AE antes, que não foram dissolvidos no momento da ETE durante a cardioversão eletiva e/ou ABL, uma vez que não foram submetidos a ETE antes da primeira prescrição do DOAC. Por fim, dada a ausência de pacientes em uso de edoxabana em nossa instituição, não é possível extrapolar essas observações para esse medicamento.

## Conclusão

A incidência de trombo atrial esquerdo e contraste espontâneo em pacientes em uso de DOAC no contexto de CVE e/ou ablação de FA, embora pequena, não é desprezível. Pacientes com escore CHA2DS2-VASc mais alto (especialmente os mais velhos) e com diâmetro do AE maior estão mais expostos a esses achados ecocardiográficos.
