# Supplementary material for: Trombo Atrial Esquerdo e Contraste Espontâneo Denso no Uso de Anticoagulante Oral de Ação Direta em Fibrilação Atrial: Visão de Centro Referenciado
Source: Arq Bras Cardiol. 2022 Sep 15;119(4):514–9. [Article in Portuguese] doi: 10.36660/abc.20210658 (PMC9563890; doi:10.36660/abc.20210658)
Supplement: Translation

# Translation: Left Atrial Thrombus and Dense Spontaneous Contrast in Direct Oral Anticoagulant Therapy of Atrial Fibrillation: Insights from a Reference Center

**Authors:** Thiago Marques, Francisco Darrieux, Fábio Gouvêa, Leandro Garambone, Ana Paula Lindoso, João Lage, Luciana Sacilotto, Ana Lúcia Coimbra, Martina Pinheiro, Natália Olivetti, Sissy Lara, Carina Hardy, Guilherme Athayde, Denise Hachul, Cristiano Pisani, Tan Chen Wu, Maurício Scanavacca

**Affiliations:** 1 Brazil Instituto do Coração, Hospital das Clínicas, Faculdade de Medicina, Universidade de São Paulo, São Paulo, SP – Brazil

**Keywords:** Atrial Fibrillation, Electric Countershock, Transesophageal Echocardiography

## Abstract

**Background:**

In the treatment of atrial fibrillation (AF), the most frequently sustained arrhythmia, with catheter ablation (CA) or electrical cardioversion (ECV), the periprocedural period is one of the most critical phases. Currently, the use of new direct action oral anticoagulants (DOAC) is increasingly frequent; however, in the real world, there are still few data on studies on the thrombus incidence in the left atrium (TrLA) or dense spontaneous contrast (DSC) on transesophageal echocardiogram (TEE).

**Objective:**

To evaluate the prevalence of events and association with risk factors in patients using DOACs. Primary objective: to analyze the prevalence of thrombus in the LA by TEE in patients using DOAC undergoing ECV/CA. Second, evaluate the association of comorbidities with the presence of thrombi and DSC.

**Methods:**

Retrospective cohort, single-center study with patients followed at the Arrhythmia Outpatient Unit (InCor-HCFMUSP). Patients indicated for procedures and using DOACs were selected, and their clinical/echocardiographic data were analyzed. A significance level of 5% was considered.

**Results:**

354 patients were included, a total of 400 procedures, from March 2012-March 2018. Thrombus in the LA was found in 11 patients (2.8%), associated with advanced age (p=0.007) and higher CHA2DS2-VASc (p<0.001) score. DSC in the LA before TEE was found in 29 patients (7.3%), with lower LVEF (p<0.038) and greater LA dimension (p<0.0001).

**Conclusion:**

The incidence of LA thrombus and DSC in patients using DOC in the context of AF ECV/CA, although small, is not negligible. Patients with higher CHA2DS2-VASc scores, especially older and with larger LA diameter, are more prone to these echocardiographic findings.

## Introduction

Atrial fibrillation (AF) is the most frequent sustained arrhythmia in clinical practice, with a prevalence of around 1% in the general population.^1^ One of the critical phases in the treatment of AF refers to the periprocedural period of catheter ablation (CA) or electrical cardioversion (ECV), where the risk of a thromboembolic event needs to be minimized with the use of oral anticoagulation. In the past, only warfarin was available, with a periprocedural incidence rate of thromboembolic events ranging between 0.5 and 1.6%.^2 , 3^ The prevalence of thrombus in the left atrium varies between 0.6% and 6.4% in patients under warfarin treatment.^4 - 6^ Currently, new direct-acting oral anticoagulants (DOACs) are increasingly frequent; however, in real-world conditions, there are still few studies with data regarding thrombus incidence or dense spontaneous contrast at TEE in the left atrium.^7 - 9^

## Methods

### Study Population

Retrospective cohort, single-center study with patients followed at the Arrhythmia Outpatient Unit of Instituto do Coração – Hospital das Clínicas da Faculdade de Medicina, Universidade de São Paulo (InCor-HCFMUSP).

Medical records of patients aged ≥ 18 years with a diagnosis of persistent AF, who underwent ECV and/or CA under the use of DOACs at least 3 weeks before the procedure, were included consecutively over 6 years (2012-2018). All patients underwent a transesophageal echocardiogram (TEE) during the same hospitalization. The choice and dose of DOAC (rivaroxaban, dabigatran or apixaban) were established by the attending physician, with the assessment of creatinine clearance during clinical follow-up. Patients who had undergone the procedure (cardioversion or ablation) more than 6 months before could be included again. Patients were constantly using DOAC, suspended only on the day of the procedure.

Clinical data such as age, comorbidities, the CHA2DS2-VASc score, structural TEE data - left atrial (LA) dimension, and left ventricular ejection fraction (LVEF) were analyzed. The diameter dimension was chosen for calculation instead of the left atrium indexed volume, as this measurement is not standard in the exam. The TEE team defined the presence of thrombi and/or dense spontaneous contrast.

## Objectives

The primary objective was to analyze the prevalence of thrombus in the left atrium, employing TEE, in patients using DOAC for at least 3 weeks who were undergoing ECV and/or ablation. The secondary objective was to evaluate the association of comorbidities with the presence of thrombi and spontaneous contrast.

This study is part of a larger register (“Registro Institucional com o uso dos Anticoagulantes de ação direta em pacientes com Fibrilação atrial não-valvar” [Institutional register for the use of direct-acting oral anticoagulants in patients with non-valvar atrial fibrillation] – CAAE # 57417716.6.0000.0068) approved by the Institutional Research Ethics Committee of the Hospital das Clínicas, University of São Paulo - CEP-HCFMUSP, approval number 1,637,837. The Ethics Committee agreed that informed consent was not required, as the study was retrospective, based on data from the institution’s medical records. All data were anonymized before being collected.

### Statistical analysis

The collected data were described as means and standard deviation values for continuous variables of normal distribution. Categorical variables were described in absolute numbers and percentages. The normality of the variables was tested using the Kolmogorov-Smirnov test. Statistical tests were performed, according to the type of variable (qualitative/quantitative) and the normality of distribution, using unpaired Student’s t-test, chi-square test and multivariate logistic regression. P-values less than 0.05 were considered statistically significant. The statistical analysis was performed using the SSPS software (version 22.0).

## Results

Three hundred fifty-four patients were included from March 2012 to March 2018. A total of 400 procedures, 122 cardioversions and 278 ablations, were performed. Among the comorbidities observed, most patients had essential hypertension, and about 33% were over 60 years old ( Table 1 ). There was a difference between the groups taking different DOACs, with a higher presence of the previous stroke in patients who used dabigatran. ( Table 2 )

**Table 1:** Population characteristics

Male (n) (%)		288 (72)
Age (SD)		59.9 (11.4)
LVEF % (SD)		59.6 (8.5)
LA mm (SD)		43.3 (6.2)
CHA2DS2-VASc (SD)		1.68 (0.25)
Hypertension (n) (%)		214 (53.6)
Stroke (n) (%)		27 (6.8)
Heart failure (n) (%)		48 (12.0)
Diabetes (n) (%)		61 (15.3)
Vasculopathy (n) (%)		26 (6.5)
DOACs	Apixaban (n) (%)	79 (19.8)
	Dabigatran (n) (%)	99 (24.8)
	Rivaroxaban (n) (%)	222 (55.5)
Dosage – Single dose (n) (%)		222 (55.5)
Thrombus (n) (%)		11 (2.8)
Spontaneous contrast (n) (%)		29 (7.3)
Thrombus and contrast (n) (%)		39 (9.8)
Procedure	Ablation (n) (%)	278 (69.5)
	Cardioversion (n) (%)	122 (30.5)

*DOAC: Direct-acting oral anticoagulants; LA: left atrium; LVEF: Left ventricular ejection fraction.*

**Table 2:** Characteristics of patients according to DOACs

	DOACs			p

	Apixaban n: 79	Dabigatran n: 99	Rivaroxaban n: 222	
Male (%)	61 (77.2)	66 (66.7)	161 (72.5)	0.29
HF (%)	5 (6.3)	15 (15.2)	28 (12.6)	0.18
Stroke (%)	5 (6.3)	13 (13.1)	9 (4.1)	**0.011**
Hypertension (%)	35 (44.3)	58 (58.6)	121 (54.5)	0.15
Diabetes (%)	8 (10.1)	18 (18.2)	35 (15.8)	0.32
Vasculopathy (%)	5 (6.3)	4 (4)	17 (7.7)	0.48
Thrombus (%)	4 (5.1)	3 (3.0)	4 (1.8)	0.31
Spontaneus contrast (%)	2 (2.5)	13 (13.1)	14 (6.3)	**0.018**

*Chi-square test. HF: heart failure ; DOAC: Direct-acting oral anticoagulant.*

There was the presence of thrombus in the left atrium in 11 patients ( Table 3 ), out of a total of 400 procedures (2.8%), and an association with advanced age (p=0.007) and higher CHA2DS2-VASc (p<0.001) was observed. ( Table 4 )

**Table 3:** Type and dosage of DOAC in patients with LA thrombus

Patient N°	Sex	Age	CHA2DS2-VASc	Rhythm in TEE	DOAC
1	Female	72	2	Irregular	Rivaroxaban 20mg
2	Female	67	3	Irregular	Dabigatran 150mg
3	Male	74	3	Irregular	Rivaroxaban 20mg
4	Male	66	1	Irregular	Apixaban 2,5mg
5	Male	64	1	Irregular	Apixaban 5mg
6	Male	67	5	Irregular	Rivaroxaban 20mg
7	Female	83	4	Irregular	Dabigatran 110mg
8	Male	58	3	Irregular	Apixaban 5mg
9	Female	78	6	Irregular	Dabigatran 110mg
10	Female	75	3	Irregular	Rivaroxaban 20mg
11	Male	56	2	Irregular	Apixaban 5mg

*TEE: transesophageal echocardiogram; DOAC: Direct-acting oral anticoagulant.*

**Table 4:** Analysis of risk factors in relation to the presence of thrombus and spontaneous contrast

	Thrombus		p	Spontaneous contrast		p	Thrombus and contrast		p
		
	Yes	No		Yes	No		Yes	No	
Male (%)	6 (2.1)	282 (97.9)	0.19	18 (6.3)	270 (93.8)	0.22	24 (8.3)	264 (91.7)	0.13
Age (SD)	69.1 (8.2)	59.6 (11.4)	**0.007**	61.7 (7.2)	59.7 (11.7)	0.19	63.5 (8.1)	59.5 (11.7)	**0.036**
HF (%)	2 (4.2)	46 (95.8)	0.52	6 (12.5)	42 (87.5)	0.14	8 (16.7)	40 (83.3)	0.09
Stroke (%)	3 (11.1)	24 (88.9)	**0.006**	6 (22.2)	21 (77.8)	**0.002**	9 (33.3)	18 (66.7)	**<0.001**
Hypertension (%)	8 (3.7)	206 (96.3)	0.20	17 (7.9)	197 (92.1)	0.57	25 (11.7)	189 (88.3)	0.16
Diabetes (%)	1 (1.6)	60 (90.4)	0.56	7 (11.5)	54 (88.5)	0.17	8 (13.1)	53 (86.9)	0.34
Vasculopathy (%)	1 (3.8)	25 (96.2)	0.72	0 (0)	26 (100)	0.14	1 (3.8)	25 (96.2)	0.29
Dosage-twice a day (%)	7 (3.9)	171 (96.1)	0.20	15 (8.4)	163 (91.6)	0.42	22 (12.4)	156 (87.6)	0.12
LA (SD)	43.6 (4.4)	43.3 (6.2)	0.86	47.8 (5.6)	43.0 (6.1)	**<0.001**	46.6 (5.6)	43.0 (6.1)	**<0.001**
LVEF (SD)	59.8 (10.0)	59.6 (8.5)	0.94	56.5 (9.0)	59.9 (8.4)	**0.038**	57.2 (9.3)	59.9 (8.4)	0.06
CHA_2_DS_2_-VASc (SD)	3.0 (1.5)	1.64 (1.3)	**0.001**	2.1 (1.4)	1.6 (1.4)	0.06	2.4 (1.5)	1.6 (1.3)	**0.001**

*Chi-square test. LA: left atrium; LVEF: left ventricle ejection fraction; HF: heart failure.*

Dense spontaneous contrast in the left atrium in the procedure prior to TEE was observed in 29 patients (7.3%). Comparing the data of this group with those without the presence of contrast, a lower LVEF (p<0.038) and a greater LA dimension (p<0.0001) were observed. ( Table 4 )

Combined findings (thrombus and contrast) occurred in 39 (9.8%) patients. In this group, older age (p = 0.007), greater LA dimension (p <0.001) and higher CHA2DS2-VASc (p <0.001) were observed. ( Table 4 )

When combining the influence of each factor by multiple logistic regression, there is a greater risk in patients with previous stroke (OR: 4.8), patients with HF (OR: 2.9), the elderly (OR: 1.04) and those with larger atrial size (OR: 1.11). An additional analysis was carried out between patients who used DOAC in a single daily dose and those who used the medication twice daily, with no difference between groups regarding thrombus, contrast, or combined situations. ( Table 05 )

**Table 5:** Multivariate analysis of risk factors

	OR	CI (95%)		p

		Inferior	Superior	
Male	0.61	0.27	1.36	0.23
HF	2.90	1.15	7.31	**0.024**
Hypertension	1.43	0.67	3.07	0.36
Age (years)	1.04	1.00	1.08	**0.045**
Stroke	4.80	1.84	12.51	**0.001**
Dosage (twice)	1.72	0.83	3.56	0.14
LA (mm)	1.11	1.04	1.17	**0.001**

*Multiple logistic regression. HF: heart failure; LA: left atrium; CI: confidence interval.*

## Discussion

In the current literature, the prevalence of thrombus in the LA among patients who were adequately anticoagulated with warfarin before TEE ranges from 0.3% to 7.7%, compared with 2.75% among other series with the use of DOACs.^10 - 14^

Our study demonstrated that, even in patients using DOACs, a rate of thrombus or dense spontaneous contrast in the LA (9,8%) was found in patients who underwent periprocedural elective ECV and/or CA. A minimum period of 3 weeks of previous use of DOACs was used, which we considered reasonable based on the literature, and there was no statistical difference compared to 4 weeks.

In a subgroup analysis of the RE-LY study, the rate of thrombi in the left atrium in patients before cardioversion was 1.5% for patients using dabigatran. In the ARISTOTLE study, TEE records were available in 86 patients using apixaban; none had a thrombus in the LA. In the ROCKET-AF study, TEE data were not collected to assess the prevalence of thrombus in the LA.^15 - 17^

Frenkel et al. retrospectively analyzed data from 388 TEE patients before AF or atrial flutter ECV using continuous DOAC or warfarin therapy for 4 weeks. Without statistical significance, the prevalence of thrombus in the LA was 4.4% in the DOAC group and 2.9% in the warfarin group.^18^ Al Rawahi et al., analyzing data from 401 patients who underwent ablation or ECV, found a thrombus in the left atrium in 11.2% of the sample. When we separated patients who used only DOACs, the presence of thrombus in those who used dabigatran, rivaroxaban and apixaban was 5%, 4% and 9%, respectively.^19^ Michael Wu et al., analyzing 609 patients using DOAC with an average anticoagulation time of 12 weeks, found 17 patients (2.8%) with thrombus in the LA and 15 patients (2.5%) with spontaneous contrast in the TEE, numbers comparable to our findings.^20^

Despite the theoretical advantages of treating AF patients with DOACs over warfarin, concerning dosage issues and subtherapeutic anticoagulation, the prevalence of thrombus in the LA is not negligible, even with the patient correctly using DOAC for at least 3 weeks. Our rates are comparable to those previously reported among patients using anticoagulation therapy with warfarin, which vary widely, as previously mentioned.

Currently, the indication to perform elective TEE before ablation and/or cardioversion of AF/atrial flutter is still controversial. According to the 2017 HRS / EHRA / ECAS Consensus on AF Catheter Ablation, 50% of the writing group members performed routine TEE, while the remaining group performed TEE only if patients had significant risk factors for LA thrombus or had not been on therapeutic anticoagulation for at least four weeks. However, it is generally accepted that the presence of a thrombus detected by TEE is a contraindication to AF catheter ablation and cardioversion.

There are several potential reasons for the presence of these findings, despite effective anticoagulation with DOAC, which include underlying factors of the patient, including severe atrial myopathy, which makes the thrombus refractory; poor adherence; inadequate dosage due to fluctuations in drug clearance (e.g., changes in renal function) and inadequate serum levels of the drug due to the incorrect mode of administration (e.g., not taking rivaroxaban with food, leading to reduced bioavailability). Of note, there was no difference between patients according to DOAC dosage regimen (bid versus qd). Another factor that can influence are drug interactions; however, in our study, there was no report of the concomitant use of drugs with high interactions already described in the literature, such as, for example, rifampicin, antiretrovirals and/or antifungals.

Our study suggests that TEE screening should be performed in patients with high CHA2DS2-VASc scores, previous ischemic stroke, and LA dimension greater than 45 mm, despite uninterrupted therapy with DOAC, a hypothesis that should be tested in future randomized clinical trials.

### Limitations

Our study has several limitations. First, this is a cross-sectional observational study. Although we excluded patients with a documented history of missed doses of DOAC in the 3 weeks prior to TEE from our study sample, it was not possible to guarantee full adherence in all individuals. In addition, we did not systematically check whether patients were taking DOAC correctly (for example, administering rivaroxaban with meal). However, our study design reflects real-world practice. Another limitation refers to the fact that it was not possible to rule out whether part of these patients already had thrombi previously in the LA that were not dissolved at the time of TEE during elective cardioversion and/or CA, since they did not undergo TEE before the first prescription of the DOAC. Finally, given the absence of patients using edoxaban in our institution, it is not possible to extrapolate these observations to this drug.

## Conclusion

The incidence of LA thrombus and spontaneous contrast in patients using DOAC in the context of AF ECV and/or ablation, although small, is not negligible. Patients with a higher CHA2DS2-VASc score (especially older ones) and a larger LA diameter are more prone to these echocardiographic findings.
